# Impact of social alienation on self-care ability in rural older adults through psychological resilience and subjective wellbeing

**DOI:** 10.3389/fpubh.2026.1748206

**Published:** 2026-01-30

**Authors:** Qi Sun, Suning Shi, Shixue Zhou, Zhaoquan Jiang

**Affiliations:** 1 The First Hospital of Jinzhou Medical University, Jinzhou, Liaoning, China; 2 Nursing Department, The 960th Hospital of the Joint Logistics Support Force of the People's Liberation Army, Jinan, Shandong, China; 3 School of Nursing, Jinzhou Medical University, Jinzhou, Liaoning, China

**Keywords:** psychological resilience, rural empty-nest older adults, self-care ability in old age, social alienation, subjective well-being

## Abstract

**Background:**

The present study is intended to examine the multiple mediating roles of psychological resilience and subjective well-being in the relationship between social alienation and self-care ability in old age among rural empty-nest older adults.

**Methods:**

From February 17, 2021, to April 20, 2023, a questionnaire survey conducted using a multistage stratifed sampling method among 425 rural empty-nest older adults (60 years and over). These participants were recruited from a rural areas of Liaoning Province, China. The questionnaire included the Social Alienation Scale, the Psychological Resilience Scale, the Subjective Well-being Scale, the Self-care Ability In Old Age Scale. A descriptive analysis was performed to characterize the sample. Linear regression was utilized to evaluate the relationship between social alienation and self-care ability in old age. PROCESS macro (Model 6) was used to analyze the multiple mediated effects of psychological resilience and subjective well-being.

**Results:**

Social alienation exerted a remarkable direct effect on the self-care ability in old age among rural empty-nest older adults (*β* = −0.678, 95% CI = −0.750– −0.607), which represented 56.45% of the total effect. Through three significantly mediated pathways indirectly affect the self-care ability in old age: (1) through the psychological resilience pathway (*β* = −0.431, 95% CI = −0.515– −0.350), which represented 35.89% of the total effect; (2) through the subjective well-being pathway (*β* = −0. 044, 95% CI = −0.089– −0.010), which represented 3.67% of the total effect; and (3) through both the psychological resilience and subjective well-being pathway (*β* = −0.048, 95% CI = −0.073– −0.029), which represented 3.99% of the total effect. The total mediating effect was 43.55%.

**Conclusion:**

Psychological resilience and subjective well-being mediate the relationship between social alienation and self-care ability in old age among rural empty-nest older adults. Therefore, healthcare professionals and stakeholders should be concerned about the psychological resilience status and mental health of rural empty-nest older adults, strengthen their attention to subjective well-being, and adopt necessary targeted intervention measures to enhance the psychological resilience and subjective well-being of rural empty-nest older adults.

## Background

China currently holds the distinction of having the world’s largest population of older adults citizens, coupled with the fastest rate of aging (1). According to the data from the seventh census in 2020, the proportion of rural residents aged 60 and above and 65 and above are 23.81 percentage points and 17.72 percentage points, respectively, which is significantly higher than in urban areas (2).

With rapid urbanization, many rural young and middle-aged laborers migrate to cities, seeking better opportunities. The white paper “The Youth of China in the New Era” released by China’s State Council Information Office in 2022 indicates that there were nearly 170 million migrant workers in 2020, most of whom are young people (3, 4).

As a result, the degree of “empty nest” is serious, and “hollow villages” are increasingly common. Rural empty-nest older adults refers to the older adults who live without their children nearby, including those without children and those living separately from their children (5–7).

This scenario presents a heavy burden for older adults care in rural China. Fully tapping the advantages and potential of rural empty nesters and improving their self-care ability in old age is of great significance for relieving the pressure of older adults care and promoting positive aging. Self-care ability in old age includes economic independence, self-care, and health maintenance (8).

Rural empty nesters often face challenges of loneliness and social alienation due to the independence of their children. Social alienation refers to a state where an individual is unable to establish positive interactions with others or the surrounding environment, leading to negative emotions (9). Social alienation can be considered a source of stress, causing psychological stress in empty nesters, and is a predictive factor for various diseases and adverse health outcomes. Recent studies have shown that social alienation is an important factor leading to loneliness and depression, which may affect the psychological health and quality of life of the older adults, thereby reducing the self-care ability of rural empty nesters (10, 11).

Recent research suggests that social alienation significantly impacts psychological resilience, defined as an individual’s ability to recover from stress, challenges, or adversity (12). Psychological resilience is a key factor in coping with life’s difficulties and is considered a vital protective factor among the older adults, helping to alleviate negative emotions and psychological stress (13, 14). For instance, prolonged social isolation can lead to persistent feelings of loneliness and emotional distress, which in turn erodes psychological resilience (15). This decline in resilience makes it more difficult for older adults individuals to recover from the stresses and challenges posed by social alienation (16).

The erosion of psychological resilience due to social alienation has a direct impact on the self-care ability of the older adults. Self-care ability involves managing one’s daily life and health, including maintaining physical health, handling finances, and performing household activities (17, 18). When older adults possess higher levels of psychological resilience, they are more likely to actively face health challenges, adapt to physical changes, and effectively manage various tasks and responsibilities in daily life. Conversely, older adults individuals with lower psychological resilience may feel more overwhelmed when facing life’s challenges, thereby impacting their self-care ability (19, 20).

Recent studies suggest a strong correlation between psychological resilience, social alienation, and self-care capabilities in older adults individuals living alone in rural areas (21). Therefore, it is possible to hypothesize that social alienation lowers the older adults’s psychological resilience, which in turn reduces self-care ability in old age. Stated differently, the association between social alienation and self-care ability in old age may be mediated by psychological resilience.

Research findings indicate that social alienation can reduce an individual’s subjective well-being, which in turn significantly impacts the self-care ability of the older adults (4). Prolonged social alienation can lead to feelings of loneliness and emotional distress, thereby decreasing individuals’ life satisfaction and happiness (22). Subjective well-being, which encompasses overall life satisfaction and emotional balance, is a crucial indicator of psychological health and quality of life (4). Higher levels of subjective well-being can enhance the confidence and positivity of older adults individuals, making them more motivated and capable of handling various challenges and tasks in life (4). Studies have shown that older adults individuals with higher subjective well-being are more proactive in dealing with health issues and daily tasks, exhibiting greater self-care abilities (23). Therefore, it can be hypothesized that social alienation affects self-care ability in old age by reducing their subjective well-being. In other words, subjective well-being may mediate the relationship between social alienation and self-care ability in old age.

Cornwell and Waite (24) pointed out that prolonged social isolation can lead to feelings of loneliness and emotional distress, which may weaken an individual’s ability to cope with life challenges, thereby reducing their level of psychological resilience. Bonanno (25) found that decreased psychological resilience makes older adults individuals more prone to negative emotions, which in turn reduces their subjective well-being.

Research findings demonstrated that older adults individuals with high psychological resilience can more effectively cope with the negative emotions brought about by social alienation, maintaining higher levels of subjective well-being (23). Ryff and Singer (26, 27) emphasized that subjective well-being is crucial for self-care ability in old age. Individuals with higher subjective well-being are more likely to proactively address health issues and daily challenges, exhibiting stronger self-care ability in old age.

This study is grounded in the Social Ecological Model (SEM) and Psychological Resilience Theory. The SEM provides a comprehensive multi-level framework, positing that individual health behaviors—such as self-care—are not merely personal choices but are shaped by a complex interplay between individual, interpersonal, and environmental factors. For rural empty-nest older adults, the SEM allows for an exploration of how distal social factors (e.g., social alienation) interact with proximal psychological resources (e.g., resilience and well-being) to determine self-care outcomes. Complementing this, Psychological Resilience Theory emphasizes the internal capacity of individuals to adapt to stressors. In the context of the “empty-nest” transition, resilience serves as a critical psychological buffer; however, chronic social alienation may erode this capacity, subsequently diminishing an individual’s motivation and ability to maintain self-care practices.

The primary objective of this study is to elucidate the mechanisms through which social alienation influences self-care ability among rural empty-nest older adults. Specifically, this research aims to: (1) quantify the direct impact of social alienation on self-care capacity; (2) examine the independent mediating roles of psychological resilience and subjective well-being; and (3) determine whether these two factors operate sequentially as a chain mediation. By identifying these pathways, the study seeks to provide a theoretical basis for targeted interventions to improve the health outcomes of this vulnerable population.

**Figure 1:**
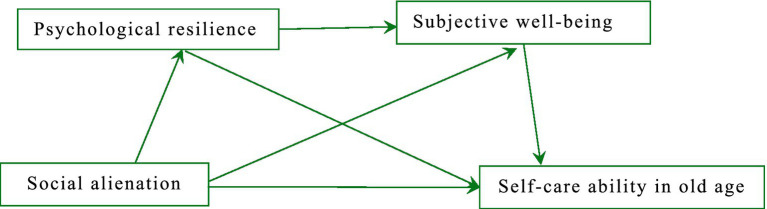
Hypothesized model.

Based on the literature review, we propose the following hypotheses (This study’s conceptual framework see Figure 1):

*H1*: Social alienation negatively impacts self-care ability in old age through psychological resilience.

*H2*: Social alienation negatively impacts self-care ability in old age through subjective well-being.

*H3*: Psychological resilience and subjective well-being jointly mediate the effect of social alienation on self-care ability in old age.

*H4*: Subjective well-being acts as a dependent variable in the relationship between social alienation and self-care ability in old age.

*H5*: A serial mediator model is more appropriate than a parallel mediator model for these relationships.

We anticipate that psychological resilience and subjective well-being can act as buffering factors, reducing the negative impact of alienation and enhancing the self-care capabilities of the older adults. Through this study, we aim to provide a theoretical basis for the formulation of targeted intervention measures to improve the quality of life for older adults people living alone, and to offer valuable information for policymakers to better support this unique group. Moreover, our research will also contribute new perspectives and data for future studies in mental health.

## Materials and methods

### Participants

Between February 17, 2021, and April 20, 2023, a multistage stratified sampling approach was employed for data collection across ten townships in Chaoyang County, Chaoyang City, located in Liaoning Province, China. A random digit table was used for all necessary calculations to select rural empty-nest older adults in Chaoyang City, Liaoning Province (a total of 8 counties) as the research population. First, the 8 counties were numbered, and 3 counties were randomly selected using a random number table (the study included Chaoyang County, Beipiao County, and Jianping County). Next, the townships in these counties were numbered as follows: Chaoyang County (25 townships), Beipiao County (27 townships), and Jianping County (24 townships). Another random number table was used to select 3 townships from each county.

For the study, older adults living alone in each selected township were systematically selected from the civil affairs department, meeting specific inclusion criteria. These criteria included willingness to participate, the ability to communicate clearly and verbally without impediments, and no documented history of neurological or psychiatric conditions.

#### Sample size and power calculation

Before data collection, a sample size calculation was performed using the G*Power Calculator to determine the appropriate sample size and ensure sufficient statistical power for the study. Based on the expected effect sizes and the desired power level (0.80) and alpha level (0.05), the calculation indicated that at least 400 participants would be required to detect statistically significant relationships between the variables. This sample size was chosen to minimize the risk of Type II errors (false negatives) and ensure the robustness of the results.

To avoid data saturation and ensure that the study had sufficient power, we aimed to distribute 450 questionnaires. In total, 425 valid responses were received, yielding a high response rate of 94.44%. Participants were registered as rural residents and permanent residents (continuously residing for ≥6 months), aged 60 or older, and were those whose children were not around for a long time (within a year, children visited for less than three months).

#### Survey process and data collection

Before conducting the survey, twenty investigators underwent standardized training to enhance their communication abilities and familiarity with the scoring system. Rural older adults living alone were approached with questionnaires. After providing informed consent, participants engaged in one-on-one interactions with the researchers and completed the surveys themselves. Throughout this process, all methods adhered to the principles outlined in the Helsinki Declaration.

#### Sample size and power calculation

To ensure statistical stringency and minimize the risk of Type II errors, an *a priori* power analysis was conducted using G*Power software (version 3.1.9.7). The calculation was based on a medium effect size ($f^2$ = 0.15), a significance level ($\alpha$) of 0.05, and a target statistical power ($1 - \beta$) of 0.80. The analysis indicated that a minimum sample size of 400 participants was required to detect significant mediating effects within the proposed structural model. To account for a potential attrition rate or incomplete responses (estimated at approximately 10–15%), the recruitment target was set at 450. Ultimately, 425 valid questionnaires were recovered, yielding an effective response rate of 94.44%, which exceeded the requirements for robust statistical inference.

#### Participants and data collection

The study focused on rural empty-nest seniors. Inclusion criteria were defined as: (1) registered rural residents with permanent residency (residing in the area for > 6 months); (2) aged 60 years or older; and (3) meeting the “empty-nest” definition (children absent for the majority of the year, with total visitation time of less than three months annually).

Data collection was executed by a team of twenty investigators who underwent standardized training to ensure inter-rater reliability, communication proficiency, and mastery of the scoring instruments. Participants were recruited through face-to-face outreach. Following the provision of informed consent, researchers administered the questionnaires via one-on-one interviews to accommodate varying literacy levels among the older participants. All procedures were conducted in strict accordance with the ethical standards of the Declaration of Helsinki.

#### Exclusion of childless older adults

To address the fundamental differences in the nature of social support, we excluded older adults without children from the study. This ensured that the focus remained on those who have children but whose children are not currently living with them, providing a consistent basis for analyzing the impact of social alienation on self-care ability.

### Measurement

#### Social Alienation Scale

The Social Alienation Scale (SAS), developed by Jessor and colleagues (28), serves as a tool to gauge individual feelings of alienation and hesitance in engaging with activities. In our research, we utilized the 15-item version of this scale, which is known for its robust validity and reliability, evidenced by a Cronbach’s alpha of 0.77. This scale is divided into four distinct subscales (29): feelings of social alienation, self-alienation, meaninglessness, and powerlessness. Responses are recorded on a four-point Likert scale, ranging from 1 (strongly disagree) to 4 (strongly agree), allowing for a total score between 15 and 60. Higher scores on this scale indicate greater social alienation. Sample items include “Sometimes I feel that the people I know are not very friendly” for social alienation and “Most of the things I do daily are very valuable and meaningful to me” for meaninglessness. In our study, the overall Cronbach’s alpha for the GSAS was 0.805, and the Cronbach’s *α* for each subscale ranged from 0.614 for powerlessness to 0.772 for feelings of self-alienation in this study. This limitation is acknowledged and discussed in the limitation section.

#### Psychological Resilience Scale

The Psychological Resilience Scale, originally developed in English by Wagnild and Young (30), was later translated into Chinese by Huang Weixiao (1). This scale comprises five dimensions: equanimity, persistence, self-reliance, meaningful life experiences, and ease of being, totaling 25 items. Each item is scored on a scale from “1” indicating complete disagreement to “7” indicating complete agreement. The total score ranges from 25 to 175, with higher scores indicating higher levels of resilience. A total score below 125 indicates a low level of psychological resilience, between 125 and 145 indicates a moderate level, and above 145 indicates a high level of resilience. Sample items include “I can get through difficult times because I’ve experienced difficulty before” for persistence and “When I make plans, I will follow through with them” for self-reliance. The scale’s Cronbach’s alpha is 0.943, with subscale Cronbach’s alphas ranging from 0.797 to 0.913.

#### Subjective Well-being Scale

The Subjective Well-being Scale, developed by Kozma et al. (31), is a tool for measuring subjective well-being, particularly in older populations. First implemented in Newfoundland in 1980, it targeted individuals aged 65–95 across urban and rural areas, as well as those in senior living facilities. The Subjective Well-being Scale is notable for its high reliability and validity (32). This scale has proven effective for assessing the subjective well-being of older adults people in China as well. Comprising 24 items, the Subjective Well-being Scale is divided into four subscales: positive affect (PA) and negative affect (NA), each with five items, and positive experience (NE) and negative experience (PE), each with seven items. The scoring system ranges from −24 to +24, with a constant of 24 added for ease of calculation, resulting in a final score range of 0–48. Higher scores indicate greater subjective well-being. According to the scale’s criteria, a total score of ≥36 suggests high subjective well-being, ≤12 indicates low subjective well-being, and scores in between reflect a medium level of well-being. Sample items include “I feel very satisfied” for positive affect and “I am in a good mood” for positive experience. In this study, the Cronbach’s *α* for the Subjective Well-being Scale’s four sub-dimensions were 0.790, 0.827, 0.746, and 0.810, respectively. Additionally, a KMO coefficient of 0.936 underscores the scale’s good reliability.

#### Self-care Ability In Old Age Scale

The Self-care Ability In Old Age Scale was developed by Pang Shuqin (33), drawing on Dorothea Orem’s theory (34) of self-care deficit within her self-care theory framework, and incorporating the Active Aging Theory (35). This Chinese scale is tailored to measure self-care ability in old age. This scale mainly consists of three dimensions: economic independence, daily living skills, and health self-maintenance, with a total of 45 items. Each item is scored from 1 (Not at all applicable) to 5(Completely applicable), with the total score ranging from 45 to 225. The higher the score, the stronger the self-care ability of the rural empty-nest older adults. Sample items include “I have adapted to the current living environment (living pace, weather, etc.)” for daily living skills and “I can maintain good interpersonal relationships” for health self-maintenance. In this study, the Cronbach’s *α* coefficient is 0.952, the Cronbach’s α for the Self-Care Ability Scale’s three dimensions were 0.876, 0.922, and 0.939, respectively. Additionally, a KMO coefficient of 0.936 underscores the scale’s good reliability. The distribution of sub-scale scores was checked to ensure they meet the assumptions required for regression analysis, and this information is included in the results section.

### Covariates

Age, gender, education level, monthly income, the contact situation between children and parents, marital status, and types of chronic diseases, were included in our study as covariates. Gender was classified into two groups: male and female. Education level was segmented into three types: elementary school or lower, middle school or less, and high school or higher. Monthly income was divided into three categories: less than 1,000 CNY, 1000 ~ 2000 CNY, and above 2000 CNY. For the contact situation between children and parents, we recognized four levels: daily contact, weekly contact, monthly contact, above monthly contact. Marital status was categorized into two: married, widow or widower. Finally, types of chronic diseases were parsed into four categories: 0 diseases, 1 disease, 2 diseases, greater than or equal to 3 diseases.

### Statistical analysis

In this study, we used IBM SPSS version 26.0 (IBM Corp, Armonk, NY) for all data analysis and processing. Initially, we performed descriptive analyses of the data, with measurement data described as means and standard deviations. Pearson correlation was then utilized to test the correlation between continuous variables. We used the PROCESS macro version 4.1 (model 6) provided by Hayes for the analysis, with the social alienation as the independent variable, Psychological resilience and subjective well-being as mediating variables, and self-care ability in old age as the dependent variable. A *p*-value of 0.05 was considered statistically significant. The study set the bootstrap confidence interval (CI) at 95% based on a bootstrap sample of 5,000. If zero was not included in the 95% confidence interval, it indicated a significant mediation effect.

## Results

### Characteristics of participants

Our study involved 425 participants, and Table 1 displays the demographic characteristics of this group. Additionally, it includes univariate analyses examining the factors influencing self-care abilities in the older adults, based on various participant traits. Out of the 425 rural empty-nest older adults, 194 (45.72%) were men and 231 (54.28%) were women. The rural empty-nest older adults’ ages ranged from 61 to 85 years, with a mean age of 70.34 ± 8.86 years. The univariate analyses of the self-care ability in old age for different characteristics are detailed in Table 1.

**Table 1:** Univariate analysis of self-care ability in old age among rural empty-nest older adults with different characteristics (*N* = 425).

Variables	Group	*N* (%)	Self-care ability in old age (Mean ± SD)	*F*/*t*	*p*
Gender	Male	194	147.07 ± 19.73	1.664	0.097
	Female	231	143.89 ± 19.49		
Education level	Elementary school or lower	123	139.54 ± 25.39	12.502	0.000
	Middle school or less	230	149.56 ± 15.11		
	High school or higher	72	141.77 ± 18.15		
Monthly income	Less than 1,000 CNY	149	139.66 ± 23.91	10.169	0.000
	1,000 ~ 2000CNY	210	148.77 ± 16.75		
	Above 2000CNY	66	147.25 ± 14.01		
The contact situation between children and parents	Daily contact	53	155.30 ± 19.67	21.814	0.000
	Weekly contact	267	147.20 ± 16.44		
	Monthly contact	65	141.46 ± 19.76		
	Above monthly contact	40	126.07 ± 25.08		
Marital status	Married	278	149.77 ± 17.07	6.720	0.000
	Widow, widower	147	136.96 ± 21.43		
Types of chronic diseases	0	104	150.42 ± 19.53	17.007	0.000
	1	141	149.27 ± 15.84		
	2	118	143.62 ± 17.42		
	Greater than or equal to 3	62	131.16 ± 24.27		

### Correlation analysis of key variables

According to the Pearson correlation study, social alienation was negatively related to self-care ability in old age and negatively related to psychological resilience and subjective well-being (*p* < 0.01). Psychological resilience exhibited a positive association with self-care ability in old age and a positive link with subjective well-being (p < 0.01); subjective well-being and self-care ability in old age had a positive correlation (*p* < 0.01). See Table 2.

**Table 2:** Descriptive statistics and correlation analysis of SAS, PRS, SWS, and SAIOAS.

Variables	Mean ± SD	1	2	3	4
SAS	40.01 ± 14.15	1			
PRS	131.33 ± 23.16	−0.506∗∗	1		
SWS	28.38 ± 12.18	−0.347∗∗	0.444∗∗	1	
SAIOAS	145.34 ± 19.64	−0.734∗∗	0.777∗∗	0.250∗∗	1

∗∗Represents *p* < 0.01.

### Analysis of chain mediation effect

We conducted a chain mediation effect analysis using the PROCESS plugin in SPSS, selecting model 6 from the chain mediation models, with 5,000 bootstrap samples. The social alienation was used as the independent variable, self-care ability in old age as the dependent variable, and sociodemographic factors as control variables. Psychological resilience and subjective well-being were included as the first and second mediating variables, respectively, in the chain mediation path. The analysis was performed on standardized data for each variable, with results detailed in Table 3.

**Table 3:** Analysis of the mediation effect of social alienation on self-care ability in old age in rural older adults after controlling for demographic variables.

Regression equation		Overall fit index			Significance	
Outcome variable	Predictors	R	R^2^	F	B	t
Psychological resilience	Social alienation	0.506	0.256	145.508	−0.827*	−12.063
Subjective well-being	Social alienation	0.466	0.217	58.532	−0.141*	−3.288
	Psychological resilience					
					0.189*	7.228
Self-care ability in old age	Social alienation	0.888	0.788	522.901	−0.678*	−18.592
	Psychological resilience				0.521*	22.319
	Subjective well-being				0.311*	7.618
Self-care ability in old age	Social alienation	0.734	0.538	492.647	−1.017*	−22.196

*p* < 0.01*, control Age, gender, education level, monthly income, the contact situation between children and parents, marital status, and types of chronic diseases.

The data showed that in the chain mediation model, social alienation negatively affected psychological resilience (*β* = −0.678, *t* = −12.063, *p* < 0.01) and negatively affected subjective well-being (*β* = −0.141, *t* = −3.288, *p* < 0.01). Psychological resilience positively affected subjective well-being (*β* = 0.189, *t* = 7.228, p < 0.01) and positively affected self-care ability in old age (*β* = 0.521, *t* = 22.319, *p* < 0.001). Subjective well-being positively affected self-care ability in old age (*β* = 0.311, *t* = 7.618, *p* < 0.01).

**Figure 2:**
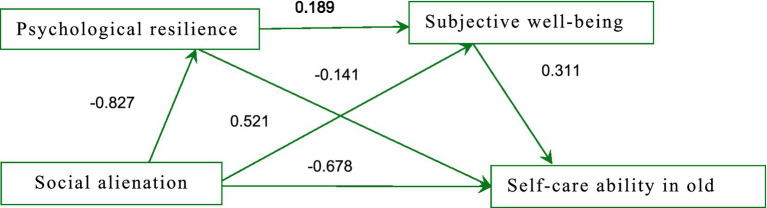
Path diagram of the impact of social alienation on self-care ability in China’s rural empty-nest older adults. The values shown are the standardized coefficients.

Using the Bootstrap test, we assessed the significance of the mediation effects through the range of the confidence intervals. In this study, the 95% confidence intervals for the chain mediation effects did not contain zero, indicating that the mediation effects were significant, as shown in Table 4. Calculating the effect sizes of each mediation path, we found that the mediation effect accounted for 43.55% of the total effect of social alienation on self-care ability in old age in rural older adults individuals, as detailed in Table 4. The path diagram is shown in Figure 2.

**Table 4:** A test of mediating effects between social alienation and self-care ability in old age.

Path	Effect value	S. E	LLCI	ULCI
Direct effect				
X → Y	−0.678	0.036	−0.750	−0.607
Intermediary effect				
X → M1 → Y	−0.431	0.041	−0.515	−0.350
X → M2 → Y	−0.044	0.019	−0.089	−0.010
X → M1 → M2 → Y	−0.048	0.011	−0.073	−0.029
Total intermediation effect	−0.523	0.042	−0.544	−0.501
Total effect	−1.201	0.045	−1.207	−0.927

X is Social alienation; M1 is Psychological resilience; M2 is Subjective well-being; Y is Self-care ability in old age.

## Discussion

In our study, we investigated how social alienation impacts the self-care abilities of the older adults living alone in rural areas. We found a strong negative association between social alienation and these individuals’ capacity for self-care. Significantly, our analysis revealed that psychological resilience and subjective well-being play a partial mediating role in this relationship.

Based on the main findings of this study, social alienation affects the self-care ability in old age among rural empty-nest older adults through the chain mediating role of psychological resilience and subjective well-being. First, studies have shown that social relationships and support are crucial for an individual’s mental health. For rural empty-nest older adults, the reduction in social support (such as children moving away and weakened community connections) leads to increased feelings of loneliness and social isolation, which may exacerbate psychological stress and, in turn, affect their mental health (36, 37). When older adults individuals lack effective contact and interaction with family, friends, and the community, their feelings of loneliness and neglect increase, leading to heightened social alienation. This prolonged social alienation not only results in mental health issues such as depression and anxiety but also affects their cognitive function and life satisfaction (38, 39), thereby reducing their self-care ability in old age.

Secondly, research has found that psychological resilience is a key factor in coping with social alienation and psychological stress. Older adults individuals with lower psychological resilience may struggle to effectively handle the emotional and behavioral issues arising from social alienation, making them more susceptible to mental health problems (40). In contrast, those with higher psychological resilience are better able to face life’s challenges with a positive attitude, thus maintaining their self-care ability despite social alienation (41).

Additionally, subjective well-being, which is an individual’s assessment of their life satisfaction, is closely related to psychological resilience and feelings of social alienation. Studies have shown that social alienation and low psychological resilience can lead to increased negative emotions and decreased life satisfaction, thereby reducing the subjective well-being of the older adults (42, 43). Older adults individuals with higher subjective well-being typically possess a more positive outlook on life and greater vitality, enabling them to participate more actively in daily activities and self-care (44).

Finally, social alienation may weaken the older adults’s experiences of autonomy, competence, and relatedness, particularly in terms of relatedness and competence, which can directly affect their self-care ability. Feeling unneeded by society or lacking opportunities for social interaction can lead to a lack of initiative and engagement in daily life, thereby impacting their self-care ability (45).

In summary, social alienation affects the self-care ability of rural empty-nest older adults by influencing their psychological resilience and subjective well-being. This chain effect underscores the importance of reducing social alienation and enhancing psychological resilience and subjective well-being. Enhancing community support and providing mental health services can effectively improve the self-care ability of this population, thereby improving their quality of life (46).

This study was dedicated to thoroughly exploring the interplay between social alienation and the capacity for self-care in older age among rural empty-nest seniors. Its aim was to provide valuable insights for the early detection and mitigation of declines in self-care abilities in this demographic, offering both theoretical and practical value. The findings highlight that addressing social isolation alone is insufficient; enhancing psychological resilience and subjective well-being is equally crucial in improving self-care capabilities.

### Key clinical implementations

#### Integrating psychological support with engaging social activities

Healthcare professionals should not only focus on addressing social isolation but also enhance psychological resilience and subjective well-being. One effective approach could be integrating psychological support with engaging social activities. Organizing regular counseling sessions led by professional therapists can help alleviate feelings of loneliness and isolation. Additionally, encouraging participation in community interest groups, craft classes, or cultural events strengthens psychological resilience and fosters a sense of belonging. This approach promotes greater life satisfaction and overall happiness among older adults.

#### Health promotion and daily life skills training

According to Positive Psychology, an individual’s well-being is closely linked to their life skills and health status. Offering health promotion initiatives, such as basic health education and daily life skills training (e.g., healthy cooking classes, simple home care skills), can greatly enhance the self-care capabilities of rural empty-nest older adults. These interventions help boost confidence, self-sufficiency, and subjective well-being, enabling older adults to manage their health more effectively and improve their quality of life.

#### Tailoring interventions for individuals with lower psychological resilience and subjective well-being

Given the significant role of psychological resilience and subjective well-being in mediating self-care abilities, healthcare providers should consider tailoring interventions and treatments for individuals with lower levels of these factors. By identifying and addressing these individuals’ specific needs, healthcare providers can improve the effectiveness of interventions aimed at enhancing self-care abilities in older adults.

#### Comprehensive strategies combining social support, mental health services, and practical skills training

A holistic approach that combines social support, mental health services, and practical skills training is essential. Implementing these strategies together can significantly improve the overall well-being and self-care capabilities of rural empty-nest older adults.

### Suggestions for further clinical practice

#### Develop multidisciplinary intervention programs

It is recommended to develop multidisciplinary intervention programs involving mental health professionals, social workers, and community organizers. These programs should work collaboratively to provide comprehensive care that addresses both the social and psychological needs of rural empty-nest older adults.

#### Monitor and evaluate the effectiveness of interventions

Ongoing monitoring and evaluation of the effectiveness of these interventions are crucial. This will help to assess improvements in self-care abilities and overall well-being, ensuring that the implemented strategies are achieving their intended outcomes.

#### Adapt interventions to local cultural and socio-economic contexts

Interventions should be tailored to account for the unique cultural and socio-economic contexts of the rural communities. This adaptability will ensure that the interventions are relevant, acceptable, and sustainable in the local setting, as recommended in the manuscript’s limitations section.

### Strengths

The main strengths of this study are as follows: first, this study was specifically designed for rural empty-nest older adults, emphasizing the important value of decreasing social alienation, improving psychological resilience and subjective well-being on the self-care ability in old age of this specific population, and providing a theoretical basis for clinicians and caregivers. Second, this study is the first to use a serial mediator model to explore the relationship between social alienation, psychological resilience, subjective well-being, and self-care ability in old age among rural empty-nest older adults. Compared with the traditional single model, this approach allowed us to consider the interactions between these factors more comprehensively.

### Limitations

#### Sample scope and generalizability

Firstly, the geographical scope of this study was confined to specific rural regions, and the sample size was relatively modest. These factors may constrain the external validity and generalizability of the results to older populations in different geographical or urban contexts. Furthermore, significant logistical challenges were encountered during the recruitment and data collection phases. Reaching older participants in remote rural areas was hindered by poor infrastructure, the digital divide, and the physical distance between households. To overcome these hurdles, researchers relied on door-to-door visits and coordination with local village committees; however, the exclusion of those with severe mobility or cognitive impairments may have introduced a degree of selection bias. Future studies should employ broader multi-regional surveys and larger cohorts to enhance representativeness.

#### Methodological and design constraints

The study’s reliance on self-reported questionnaire surveys may introduce subjective biases, such as social desirability bias or recall bias. Although questionnaires are efficient for quantitative analysis, they may not capture the nuanced lived experiences of the participants. Future research would benefit from a mixed-methods approach, incorporating in-depth interviews and case studies to provide a more comprehensive qualitative understanding. Additionally, as this was a cross-sectional study, it captures only a snapshot in time, precluding the ability to establish causal relationships or observe long-term psychological trajectories. We recommend that future researchers adopt a longitudinal design to track the evolution of psychological states over time.

#### COVID-19 context and precautions

Data collection for this study occurred continuously throughout the COVID-19 pandemic. While this provides a unique reflection of the ongoing psychological impact of a global health crisis, the results may be influenced by pandemic-specific stressors—such as social isolation, fear of infection, and disrupted healthcare access—that may not be present in a post-pandemic landscape. To ensure the safety of the participants and the research team, strict COVID-19 precautions were implemented during all face-to-face interactions. These included the mandatory use of N95 masks, maintaining a physical distance of at least two meters, conducting interviews in well-ventilated outdoor spaces when possible, and performing health screenings (temperature checks) prior to engagement. These measures, while necessary, may have subtly influenced the rapport between researchers and participants.

#### Measurement reliability

The reliability of the Social Alienation Scale used in this study warrants caution. The Cronbach’s $\alpha$ coefficients for the subscales ranged from 0.614 (powerlessness) to 0.772 (self-alienation). Specifically, the relatively low reliability of the powerlessness subscale may affect the robustness of the statistical outcomes. Future studies should consider utilizing scales with higher internal consistency or adapting existing instruments to better fit the linguistic and cognitive profiles of rural older adults to ensure more accurate measurement.

#### Cultural context and confounding variables

This study did not fully account for certain confounding variables, such as detailed socio-economic status and deep-seated cultural backgrounds. In the rural setting of this study, traditional values of filial piety significantly influence elder care practices. There remains a strong cultural preference for home-based care provided by kin; consequently, nursing homes are often viewed with social stigma and are perceived as a “last resort” for those abandoned by their families. This cultural landscape likely exacerbates the feelings of alienation among those living alone. Future research should integrate these cultural constructs and socio-economic indicators to explore how they moderate the relationship between living arrangements and psychological well-being.

## Conclusion

The primary objective of this study was to elucidate the complex mechanisms through which social alienation influences the capacity for self-care among rural empty-nest older adults, specifically examining the mediating roles of psychological resilience and subjective well-being. The findings demonstrate that social alienation exerts a significant negative impact on self-care capacity. This relationship is not direct but is partially explained by the parallel and sequential mediation of psychological resilience and subjective well-being. Collectively, these variables form a critical pathway that determines the functional independence of the older adults in rural settings.

The results underscore the vulnerability of rural empty-nesters, whose diminished self-care abilities are exacerbated by social isolation and psychological distress. Consequently, it is imperative to implement multi-dimensional strategies that transcend basic physical care. Healthcare providers, local government agencies, and community stakeholders should prioritize interventions that bolster psychological resilience and enhance subjective well-being. By fostering social connectivity and psychological strength, it is possible to mitigate the deleterious effects of alienation on functional health.

Furthermore, this study highlights the necessity for tailored support systems. Interventions should be specifically designed for individuals identified as having low levels of resilience or poor life satisfaction. Future research should build upon these narrative findings by employing longitudinal designs to evaluate the long-term efficacy of such psychological interventions. Ultimately, addressing the psychological precursors of self-care capacity is essential for promoting healthy aging and improving the quality of life for the growing population of rural older adults living alone.

## Data Availability

The raw data supporting the conclusions of this article will be made available by the authors, without undue reservation.
